# Computer-Aided Design and 3D Printing of Hemipelvic Endoprosthesis for Personalized Limb-Salvage Reconstruction after Periacetabular Tumor Resection

**DOI:** 10.3390/bioengineering9080400

**Published:** 2022-08-18

**Authors:** Xianglin Hu, Yong Chen, Weiluo Cai, Mo Cheng, Wangjun Yan, Wending Huang

**Affiliations:** 1 Department of Musculoskeletal Surgery, Fudan University Shanghai Cancer Center, Shanghai 200032, China; 2 Department of Oncology, Shanghai Medical College, Fudan University, Shanghai 200032, China

**Keywords:** 3D-printing, periacetabular tumor, surgery, pelvic reconstruction, hemipelvic prosthesis, personalized medicine

## Abstract

3D-printed hemipelvic endoprosthesis is an emerging solution for personalized limb-salvage reconstruction after periacetabular tumor resection. Further clinical studies are still required to report its surgical characteristics, outcomes, benefits and drawbacks. Sixteen consecutive patients underwent periacetabular tumor wide resection and pelvic reconstruction with a 3D-printed hemipelvic endoprosthesis from 2018 to 2021. The surgical characteristics and outcomes are described. The mean follow-up duration was 17.75 months (range, 6 to 46 months). Five patients underwent surgery for type I + II resection and reconstruction, seven for type II + III resection and reconstruction, three for type II resection and reconstruction, and one for type I + II + IV resection and reconstruction. The incidence of postoperative complication was 12.5% (2/16) for deep venous thrombosis (DVT), 12.5% (2/16) for pneumonia, and 12.5% (2/16) for would deep or superficial infection. During follow-up, two patients (12.5%) suffered hip dislocation and underwent revision surgery. CT demonstrated an obvious prosthetic porous structure–bone fusion after follow-up of at least 6 months. At the final follow-up, 12 lived with no evidence of disease while four lived with disease; no patients experienced pain; and 15 had independent ambulation, with a mean Musculoskeletal Tumor Society (MSTS) score of 85.8% (range, 26.7% to 100%). 3D-printed hemipelvic endoprosthesis facilitates wide resection of periacetabular tumor and limb-salvage reconstruction, thus resulting in good oncological and functional outcomes. The custom-made nature is able to well mimic the skeletal anatomy and microstructure and promote osseointegration. Perioperative complications and rehabilitation exercise still need to be stressed for this engineering technology-assisted major orthopedic surgery.

## 1. Introduction

The pelvis is the most common anatomic site at which malignant bone tumors occur, whether primary or metastatic. It is reported that approximately 16.2% of primary bone sarcomas are located in the pelvis, e.g., pelvic chondrosarcoma, osteosarcoma, or Ewing tumor, causing a poor five-year survival rate of 45.0~57.5% [1]. The pelvic bone is also a common destination of many major metastatic cancers, such as lung and breast cancers [2]. Based on the Enneking and Dunham classification [3], pelvic tumors are generally divided into four types on the basis of the anatomic sites involved: type I involves the iliac bone; type II involves the acetabulum; type III involves the pubis and the ischium; and type IV involves the ilium to sacrum. Of the four types, periacetabular tumor, namely type II, remains the most debilitating and technically demanding in surgical treatment [4]. Periacetabular tumors can cause refractory pain, limit hip joint autonomy, and make walking difficult, thus causing a serious damage to the patient’s quality of life and survival [5]. Wide resection of periacetabular tumors with a negative surgical margin is the most effective way to locally control the tumor and improve survival [6,7,8]; however, this makes reconstruction challenging, requiring recovery of hip joint function, maintenance of pelvic support, and reservation of lower limb length and function [9,10].

In recent decades, many reconstructive techniques have been developed for acetabular tumor resection, including saddle prosthesis, modified Harrington procedure, modular hemipelvic endoprosthesis, and so on [9,10]. In recent years, with the rise of addictive manufacturing, 3D-printed custom-made hemipelvic endoprostheses are increasingly popular in acetabular reconstruction after oncological resection [11]. 3D-printed orthopedic implants have a free-forming advantage, which can solve the shape-mismatching problem of traditional universal implantable devices; they are compatible with the body’s own bone both in terms of shape and mechanical properties. In addition, 3D printing process technology can integrate dense parts and porous structures to promote osteoblast adhesion and autologous bone ingrowth [12,13,14]. As an emerging technique with unique advantages in periacetabular reconstruction, to date, 3D-printed hemipelvic endoprosthesis implantation can only be performed in some large tertiary hospitals, and adequate explorations on its benefits and drawbacks are still lacking. In this clinical case study, we aimed to share our institutional surgical experience of 3D-printed hemipelvic endoprosthesis reconstruction after wide resection of a periacetabular tumor. This study may prompt more orthopedic oncologists to attempt to better understand this emerging reconstructive technique.

## 2. Patients and Methods

### 2.1. Patients

Between August 2018 and December 2021, a total of 16 patients diagnosed with a periacetabular tumor underwent tumor wide resection and hemipelvic reconstruction with a 3D-printed endoprosthesis in the Department of Musculoskeletal Surgery of Fudan University Shanghai Cancer Center, Shanghai, China. There were 8 males and 8 females, with a mean age of 42.8 years (range, 19 to 67 years). The mean body mass index (BMI) was 24.2 kg/m^2^ (range, 15.9 to 33.3 kg/m^2^). All patients complained of unilateral hip pain and 5 had hip motion limitation causing difficulty in walking. Pain was assessed using a visual analogue scale (VAS) [15], with a mean score of 6.4 (range, 4 to 9). All patients had a preoperative bone biopsy confirming a malignant or benign aggressive tumor, including 8 primary bone sarcomas (4 chondrosarcoma, 2 Ewing tumor, 1 osteosarcoma and 1 fibrosarcoma), 2 epithelioid hemangioendothelioma, 1 tendon sheath giant cell tumor and 5 metastatic cancers. On the basis of the Enneking and Dunham classification [3], 7 patients had tumors of II + III type, 3 had II type, 5 had I + II type, and 1 had I + II + IV type. Six patients had a surgical history of primary lesion. Eleven patients received neoadjuvant oncological therapy before surgery. The patients’ demographic and clinical characteristics are detailed in Table 1. This study was approved by the review board of our institution. All patients provided written informed consent.

### 2.2. Hemipelvic Endoprosthesis Design and Manufacture

Our workflow for designing and manufacturing the 3D-printed custom-made hemipelvic endoprosthesis included three main steps. First, imaging reconstruction of patient’s pelvis and lower limbs. After hospital admission, all patients were subjected to X-ray, thin-slice computed tomography (CT), and magnetic resonance (MR) of the lesion. The treating surgeon raised a 3D-printed prosthesis requirement and exported the patient-specific CT and MR data (DICOME format) to the engineer. The CT and MR data were then imported into the Materialise Mimics software package, where a 3D pelvic model was reconstructed and simplified by means of automatic generation and manual segmentation. The 3D model was exported in STL format. Skeleton-related data were measured. The CT and MR data were generally acquired before initiation of tumor neoadjuvant therapy.

Second, prosthesis design and structure optimization was carried out. Osteotomy was simulated on the basis of the location of the tumor in the 3D model using the Materialise Mimics software. The 3D model, which was exported in STL format, was further processed using the Geomagic Studio software package. An IGS formatted data file was generated. The patient-specific prosthesis was designed and optimized with the fine structure in the CAD software (CREO). The treating surgeon and the engineer collectively determined the tumor resection border and osteotomy position through consultation. A surgical guide template was also designed using the CAD software based on the osteotomy site. The designed skeletal model, prosthesis model, and surgical guide template model were imported into Materialise Mimics, where resection and reconstruction surgery were simulated to test the rationality of those models.

Third, the titanium metal prosthesis was 3D printed. The determined skeletal and prosthesis models were imported in STL format into the Arcam Q10 Plus 3D printer. Ti_6_Al_4_V powder was selected as the material with which to print the prosthesis. Electron beam melting (EBM) manufactures parts by melting metal powders layer by layer using a magnon-conduction electron beam under a high vacuum. EBM is used both for printing and as a source of energy for heating. Preheating is used to sinter the powder and raise the powder temperature (to 600~1200 °C). Two different types of melting, contour and hatch, can be used. Optimization and compensation functions were applied. The finished metal prosthesis was polished and measured to confirm that the size was appropriate. A matched surgical guiding plate for osteotomy was also printed using the PLA material. Generally, the process took two weeks from imaging acquisition to finished prosthesis.

### 2.3. Surgery and Follow-Up

After general anesthesia, the patient was placed in a lateral position. The posterior iliac approach was adopted in combination with the Smith–Petersen approach. If the tumor involved the type III zone, an additional inguinal incision was performed. After incision, the abdominal muscles were separated from the iliac crest to the medial level of the sacroiliac joint. The iliopsoas muscle, iliac vessels, and femoral nerve were carefully protected. The gluteus muscle was separated to the level of the posterior superior iliac spine and greater sciatic foramen, with the distal exposure of the femur lesser trochanter. The starting and ending points of the muscles around the hip joint were preserved as much as possible. The soft tissue margin of the tumor was ensured. The femoral head was resected to better expose the pelvic structure and tumor tissue. Based on the osteotomy location determined by the preoperative plan, the osteotomy guide template was used, and the osteotomy was performed with an ultrasonic bone knife or a special electric saw. The accuracy of osteotomy was tested with the 3D-printed prosthesis. The 3D-printed prosthesis was installed and fixed as planned. The femoral stem prosthesis was installed. Then, we recovered and checked the hip joint posture to minimize its dislocation risk. The abdominal muscle and gluteus muscle were sutured and fixed on the prosthesis. Drainage tubes were placed. The muscles and ligaments around the artificial hip joint were reconstructed as much as possible.

From one to seven days post operation, subcutaneous injection of low molecular heparin was administrated to prevent deep venous thrombosis (DVT). Additionally, early rehabilitation training on the bed was recommended for three weeks post operation, including functional training of the ankle and hip joints and quadriceps femoris strength training. After three weeks post operation, it was recommended that patients perform off-bed activities with a walking aid or stick, and gradually perform independent walking.

Surgical type, duration, blood loss, postoperative complication and VAS score at the 7th day post surgery were recorded. The surgical margin reported by postoperative pathology was recorded. Post-discharge follow-up was conducted every three months for one year, and then once a year after one year. Follow-up items included need for revision surgery, adjuvant therapy, oncological outcome, functional outcome, radiological examination, VAS, and Musculoskeletal Tumor Society (MSTS)-93 score [16]. Follow-up was conducted until 20 June 2022. The mean follow-up duration was 17.75 months (range, 6 to 46 months, Table 2).

### 2.4. Statistical Analysis

Data are presented mean with range or as a number with percentage. All statistical analysis is descriptive and was conducted using SPSS 26.0.

## 3. Results

**Figure 1:**
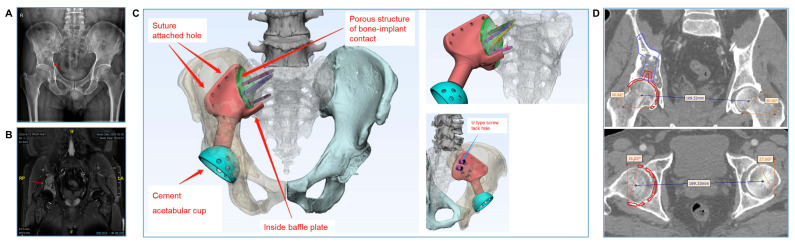
**Case 2. Prosthesis design and installation simulation.** (**A**,**B**) Preoperative X-ray and MR displayed the tumor involving the right acetabulum and ilium (red arrow). We planned to totally remove the right iliac bone from the right sacroiliac joint position. (**C**) Design of the 3D-printed hemipelvic endoprosthesis. The prosthesis is modular, and consists of an iliac holder (red) and an acetabular cup (blue). Between the iliac holder prosthesis and the autologous bone surface, a porous structure with a size of 2.5 mm mimicking the trabecular bone was designed (green) to promote bone ingrowth and fusion. In the sacroiliac joint, the design included four screws (icons 1, 2, 3 and 4), bypassing the sacral canal and knocked into the anterior side of the sacrum. Of these, screws 1, 2, and 3 are knocked into S1, while screw 4 is knocked into S2. Two U-shaped screw track holes are included at the rear of the prosthesis with which to fix the nail rod system. (**D**) Simulated installation of the 3D-printed acetabular cup; the abduction angle is about 40°, and the anteversion angle is about 16°.

**Figure 2:**
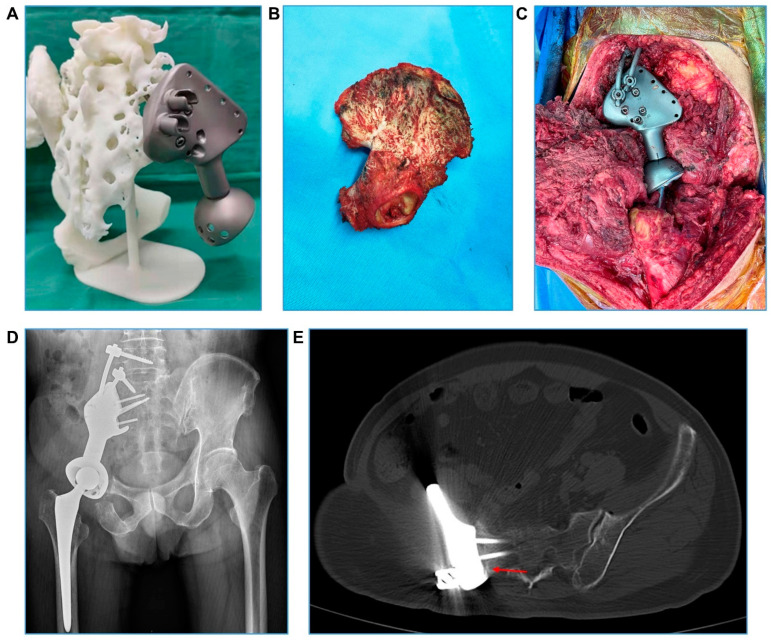
**Case 2. Intra- and post-operative images.** (**A**) Finished 3D-printed hemipelvic endoprosthesis. (**B**) The total en bloc removed ilium and acetabulum. (**C**) The 3D-printed prosthesis is installed and fixed. (**D**) X-ray shows a good implantation effect at the visit 6 months post operation. (**E**) CT shows good bone ingrowth and fusion at the prosthesis and bone interface (red arrow) at the visit 10 months post operation.

**Figure 3:**
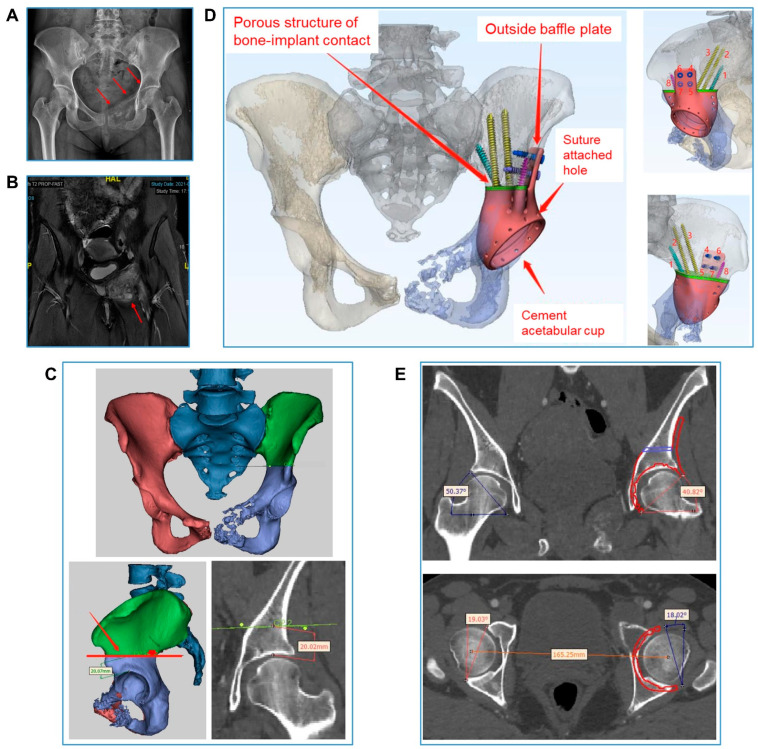
**Case 3. Prosthesis design and installation simulation.** (**A**,**B**) Preoperative X-ray and MR displaying the tumor, involving the left acetabulum, the pubis, and the ischium (red arrow). (**C**) Simulation of osteotomy: the osteotomy line is about 20 mm from the upper margin of the acetabulum. (**D**) Design of the 3D-printed hemipelvic endoprosthesis, which is integrative. Between the prosthesis and the autologous bone surface, a porous structure was designed with a size of 3.0 mm mimicking the trabecular bone (green) for the promotion of bone ingrowth and fusion. Screws 1, 2, and 3 are in the iliac force line direction (length, 60 to 80 mm). Screws 4, 5, 6 and 7 are in the lateral direction (length, 20 to 25 mm). Screw 8 is a short spike in the anterior column of the ilium. (**E**) Installation simulation of the 3D-printed acetabular cup: the abduction angle is about 40° and the anteversion angle is about 18°.

**Figure 4:**
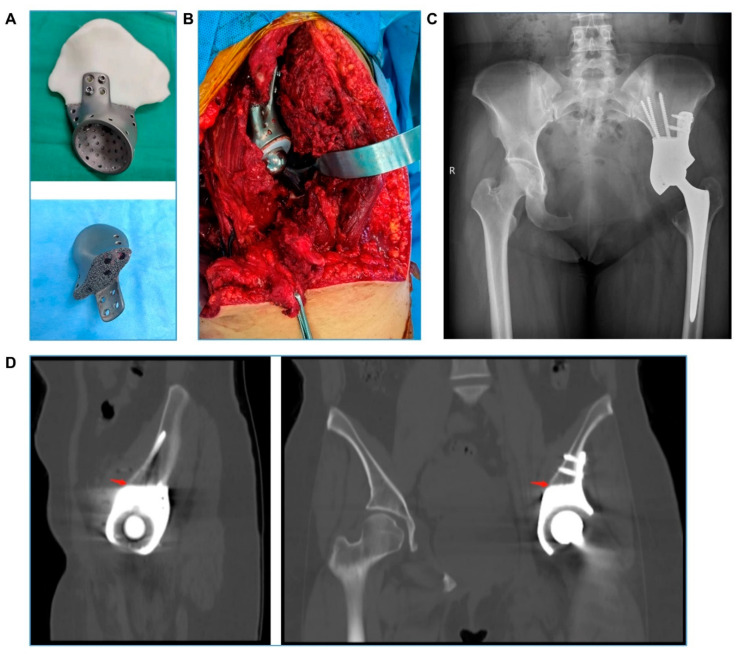
**Case 3. Intra- and post-operative images.** (**A**) Finished 3D-printed prosthesis: The porous side is clearly shown. (**B**) The 3D-printed prosthesis is installed and fixed. (**C**) X-ray shows a good implantation effect at the visit 3 months post operation. (**D**) CT shows good bone ingrowth and fusion at the prosthesis and bone interface (red arrow) at the visit 10 months post operation.

Five patients underwent surgery for type I + II resection and reconstruction, seven for type II + III resection and reconstruction, three for type II resection and reconstruction, and one for type I + II + IV resection and reconstruction. A representative type I + II resection and reconstruction (case 2) is detailed in Figure 1 and Figure 2; a representative type II + III resection and reconstruction (case 3) is detailed in Figure 3 and Figure 4, including the prosthesis design, installation, and follow-up imaging.

The surgery characteristics and follow-up outcomes are detailed in Table 2. The mean surgery time was 289.7 min (range, 200 to 390 min). The mean blood loss was 1563 mL (range, 400 to 3600 mL). All patients had a negative postoperative pathological margin. Surgical margin was on average 20.6 mm (range, 10~33 mm) away from the tumor margin. The incidence of postoperative complication was 12.5% (2/16) for DVT, 12.5% (2/16) for pneumonia, and 12.5% (2/16) for the occurrence of deep or superficial infection. The patient with deep infection (Case 8) was cured by means of debridement. The patient’s pain was well resolved by the 7th day post operation, with a mean score of 0.9 (range, 0 to 3). All patients recovered and were discharged. During follow-up, two patients (12.5%) experienced hip dislocation and underwent revision surgery, and eight patients received adjuvant oncological therapy. Fourteen patients were followed up for at least six months, all of whom presented evident prosthesis–bone fusion upon CT imaging. At the end of follow-up, 12 lived with no evidence of disease, while four lived with disease; no patients experienced pain; 15 had independent ambulation, with the exception of Case 5; the mean Musculoskeletal Tumor Society (MSTS) score was 85.8% (range, 26.7% to 100%).

## 4. Discussion

Within the last ten years, 3D printing technology has increasingly been applied in hip and pelvic surgery, mainly in China and the USA [17,18,19]. In our medical center, we generally use this technology for: (1) 3D-pirnted hemipelvic endoprosthesis and instruments for implantation; (2) 3D-printed patient-specific 1:1 pelvic anatomical models for preoperative planning and surgery simulation (Figure 2A); and (3) 3D-printed surgical guiding plates for precise intraoperative osteotomy (Supplementary Figure S1). In China, Liang et al. [17] first demonstrated that 3D-printed pelvic prosthesis was feasible and safe to perform reconstruction following pelvic malignant tumor resection on the basis of 35 cases between 2013 and 2015. Compared to Liang and colleagues’ [20] study, in which wide resection was only performed in 15 of the 35 patients, our study conducted wide and precise resection of all tumors, achieved as a result of sufficient preoperative planning and the intraoperative utilization of a 3D-printed osteotomy guiding plate. Both 3D-printed models for preoperative planning and 3D-printed patient-specific instrumentation (PSI) for use during the operation can improve the accuracy of osteotomy [21,22]. The surgical boundary for musculoskeletal tumors is usually determined based on the MSTS/Enneking system, an includes intracapsular, marginal, wide, or radical resection. Considering the anatomical specificity of the pelvis, radical resection is rarely performed. We determined whether the boundary of the tumor resection was safe on the basis of a comprehensive consideration of the distance of the compartment, barrier, and tumor edge. The distance between the osteotomy level and the tumor should be 10–30 mm for primary malignant tumors, and 10–20 mm for metastatic tumors.

In the working process of our team, the orthopedic surgeons first indicate the optimal osteotomy position that will ensure an adequate margin, e.g., the total removal of the right iliac bone in Case 2, and 20 mm from the upper margin of the left acetabulum in Case 3, on a patient-by-patient basis. Then, the engineer will design a custom-made hemipelvic endoprosthesis to reconstruct the bone defect based on the simulated osteotomy. Under the conditions of this reconstructive security, the orthopedic surgeons are able to radically resect the malignant tumor to the greatest degree possible. Xu et al. [23] conducted a comparative study between 3D-printed prosthetic and conventional reconstructions after pelvic tumor resection, finding that patients with 3D-printed prosthetic reconstructions were more likely to have a negative resection margin compared to those with conventional reconstruction (9/10 versus 3/10). In our concept, and on the basis of our institutional surgical experience, the resection extent is determined by the degree of tumor malignancy: the surgical margin needs to be at least 2 cm away from G1~2 primary bone tumors, 3 cm away from G3 primary bone tumors and 2 cm away in the case of metastatic bone disease.

Considering the anatomical and functional complexities of the hip joint, 3D printing technology is particularly advantageous in periacetabular tumor resection and reconstruction [24,25,26,27]. However, the surgical characteristics and outcomes vary from hospital to hospital, and there is a lack of an associated standard of application. As shown in Table 3, our institution had similar surgical durations, but relatively less blood loss compared to other institutions. The pelvis is rich in blood vessels, and massive bleeding often occurs during surgery [25,26,27]. Control of bleeding is one of the most important issues in pelvic surgery. In addition to careful and skilled intraoperative manipulation, measures for reducing bleeding include controllable low-blood-pressure anesthesia, trans-catheter tumor arterial embolization, temporary occlusion of the iliac artery, and temporary abdominal aortic balloon occlusion. Recently, Tripathi et al. [28] designed a 3D composite scaffold based on bioprinting technology and the synergistic hemostasis mechanisms of cellulose nanofibrils, chitosan, and casein in order to control blood loss during traumatic hemorrhage and accelerate wound healing. In the future, 3D printing technology using “bioinks” may offer the desired hemostasis activity and wound-healing effect [28,29]. These prospects will be a major breakthrough, upgrading current 3D-printed pelvic implant surgical procedures, in which excessive bleeding and delayed wound healing are common (Table 3).

Our study demonstrated good osseointegration, which can be attributed to the designed porous structure of 3D-printed prosthesis. This biological fusion may provide patients with long-term prosthesis stability when compared to a conventional prosthesis. In our study, the material used for the prosthesis was titanium alloy (Ti_6_Al_4_V). In recent years, 3D-printed biomaterials and tissue engineering scaffolds with osteoinductive properties have been explored, showing promise for the further improvement of osseointegration in clinical applications [30]. Sometimes, deep infection occurred in those cases undergoing 3D-printed prosthesis reconstruction (Table 3). This requires orthopedic surgeons to minimize the duration of surgery, use antibiotics appropriately, and sometimes to perform a debridement in a timely fashion. 3D-printed bone scaffolds with anti-bacterial activity seem to be a good option for preventing this situation in the future [31]. In addition, sometimes, marginal resection is inevitable in pelvic tumor surgery as a result of the tumor anatomic site and size. Some material scientists have proposed that, for bone tumor treatment, the biomaterials should possess a dual function of killing tumor cells and regenerating bone defects simultaneously. Therefore, 3D-printed scaffolds with anti-tumor bioactivity have been rapidly researched and developed in recent years with the aim of killing residual tumor cells [32,33]. On the other hand, the surgical techniques used in 3D-printed prostheses for periacetabular reconstruction have also been the subject of continuous innovation [34,35,36].

There are still some limitations. To date, most studies, including ours, related to 3D-printed prostheses for periacetabular reconstruction are based on single-center case series, with a low number of patients. Some patients may be precluded from benefiting from this surgery due to its high cost, which is not covered in the national medical insurance reimbursement catalogue. Secondly, no more than ten years have elapsed between the first application of this surgery and today; the median follow-up time reported by previous studies did not exceed three years. The long-term stability, functional outcomes, and lifespan of the 3D-printed hemipelvic endoprosthesis remain unclear. Thirdly, the waiting time from prosthesis design to the final product still needs to be minimized. A promising direction would be to establish an online modular database to assist in the rapid design of prostheses.

In summary, this study presents our institutional experience regarding 3D-printed prostheses for limb-salvage reconstruction following periacetabular tumor resection. The 3D-printed hemipelvic endoprosthesis facilitates wide resection of periacetabular tumors and limb-salvage reconstruction, thus resulting in good oncological and functional outcomes. In the future, continuous innovations in terms of both surgical techniques and prosthesis materials will further improve this major engineering technology-assisted orthopedic surgery for patients with periacetabular tumor.

## Figures and Tables

**Table 1:** Demographic and clinical characteristics of the enrolled patients.

Case	Age (Years)	Sex	BMI (kg/m^2^)	Initial Symptoms and Signs	VAS at Admission	Disease Course (Months)	Tumor Characteristics				Surgical History	Neoadjuvant Oncological Therapy
							Diagnosis	Side	Zone	Stage *		
1	32	F	21.6	Hip pain, limitation of motion	6	20	Recurrent fibrosarcoma	L	II	IIB	Piecemeal resection	Chemotherapy
2	67	M	24.8	Hip pain	7	48	Chondrosarcoma	R	I–II	IIB	/	/
3	31	F	26.2	Hip pain	5	3	Chondrosarcoma	L	II–III	IIB	/	/
4	41	M	20.5	Hip pain	6	15	Chondrosarcoma	R	I–II	IIB	/	/
5	32	M	28.7	Hip pain, limitation of motion	6	7	Tendon sheaths giant cell tumor	L	II–III	3	/	Denosumab
6	44	F	21.5	Hip pain	5	6	Epithelioid Hemangioendothelioma	R	II–III	IB	/	/
7	19	M	15.9	Hip pain	7	2	Osteosarcoma	R	I–II	IIIB	/	Chemotherapy
8	38	F	22.9	Hip pain	4	3	Epithelioid Hemangioendothelioma	L	I–II	IB	/	Radiotherapy
9	58	F	22.3	Hip pain	7	5	Chondrosarcoma	L	II–III	IIB	/	/
10	22	M	27.4	Hip pain, limitation of motion	8	10	Ewing sarcoma	R	I–II	IIIB	/	Chemotherapy, Radiotherapy
11	23	F	33.3	Hip pain	7	7	Ewing sarcoma	L	I–II, IV	IIIB	/	Chemotherapy
12	47	F	23.7	Hip pain, limitation of motion	8	5	Metastatic lung adenocarcinoma	R	II–III	T2N0M1	Primary tumor surgery	Chemotherapy
13	60	M	27.4	Hip pain, limitation of motion	8	6	Metastatic renal clear cell cancer	L	II–III	T1N0M1	Primary tumor surgery	Targeted therapy, Immunotherapy, Radiotherapy
14	57	M	25.7	Hip pain	9	7	Metastatic renal clear cell cancer	L	II–III	T1N0M1	Primary tumor surgery	Targeted therapy
15	60	F	21.3	Hip pain	5	2	Metastatic cyst-adenocarcinoma of the submandibular gland	R	II	T1N0M1	Primary tumor surgery	Chemotherapy
16	53	M	23.9	Hip pain	5	1	Metastatic hepatocellular cancer	L	II	T1N0M1	Primary tumor surgery	Targeted therapy, Immunotherapy

F: female; M: male; BMI: body mass index; VAS: visual analogue scale; R: right; L: left. * Stage: Enneking stage for primary tumor and TNM stage for metastatic tumor.

**Table 2:** Surgery characteristics and outcomes.

Case	Resection Type *	Surgery Time (min)	Blood Loss (mL)	Surgical Margin		Perioperative Complication	VAS at 7th Post-Surgery Day	Post-Discharge Follow-Up					
				Distance (mm)	Pathology			Time (Month)	Revision Surgery	Adjuvant Therapy	Oncological Outcome	VAS	MSTS Score (%)
1	II	205	400	33	Negative	/	0	9	/	/	No evidence of disease	0	27 (90%)
2	I–II	325	700	25	Negative	Pneumonia	0	13	/	/	No evidence of disease	0	27 (90%)
3	II–III	365	800	20	Negative	/	0	13	/	/	No evidence of disease	0	29 (96.7%)
4	I–II	305	1100	20	Negative	/	1	39	Done for hip dislocation caused by a traffic accident at 17th month	/	No evidence of disease	0	30 (100%)
5	II–III	320	3600	25	Negative	DVT	3	14	/	/	No evidence of disease	0	8 (26.7%)
6	II–III	330	3000	26	Negative	/	0	10	/	/	No evidence of disease	0	27 (90%)
7	I–II	215	500	14	Negative	/	2	18	/	Chemotherapy	Alive with disease	0	26 (86.7%)
8	I–II	340	1900	21	Negative	DVT, deep infection	0	17	/	/	No evidence of disease	0	28 (93.3%)
9	II–III	350	3200	25	Negative	/	1	10	/	/	No evidence of disease	0	27 (90%)
10	I–II	230	2000	20	Negative	Superficial infection	2	9	/	Chemotherapy	Alive with disease	0	26 (86.7%)
11	I–II, IV	260	1300	10	Negative	/	1	38	/	Chemotherapy	Alive with disease	0	27 (90%)
12	II–III	210	900	20	Negative	/	1	6	/	Chemotherapy, Bisphosphonates	No evidence of disease	0	27 (90%)
13	II–III	380	1600	18	Negative	/	2	7	Done for hip dislocation at 3rd month	Targeted therapy, Immunotherapy, Denosumab	No evidence of disease	0	23 (76.7%)
14	II–III	390	2600	16	Negative	Pneumonia	1	12	/	Targeted therapy, Bisphosphonates	No evidence of disease	0	27 (90%)
15	II	200	600	20	Negative	/	0	46	/	Chemotherapy, Bisphosphonates	Alive with disease	0	28 (93.3%)
16	II	210	800	17	Negative	/	1	23	/	Targeted therapy, Denosumab	No evidence of disease	0	25 (83.3%)

F: female; M: male; BMI: body mass index; VAS: visual analogue scale; DVT: deep venous thrombosis; MSTS: Musculoskeletal Tumor Society. * Based on the Enneking and Dunham classification.

**Table 3:** Surgical characteristics and outcomes of 3D-printed prosthesis reconstruction after periacetabular tumor resection reported by large tertiary hospitals in China.

Reference, Publication Year and Journal	Institution	Study Period	Patient Number	Age (Years)	Sex (Male /Female)	Resection Type	Surgical Duration (min)	Blood Loss (mL)	Surgical Margin	Complication	Follow-Up Months	MSTS (%)
Ji et al. [24], 2020, J Bone Joint Surg Am	People’s Hospital, Peking University	2015~2017	80	41.9 (11~78)	42/38	II (*n* = 23) II + III (*n* = 57)	276 (150~570)	1898.5 (300 to 6000)	R0 for 61 of 64 primary tumors; R1 for 16 metastatic tumors	Wound dehiscence (*n* = 8) Deep infection (*n* = 5) Hip dislocation (*n* = 2) Hematoma (*n* = 2) Acute arterial thrombosis (*n* = 1) Screw breakage (*n* = 1)	32.5 (9~52)	83.9% (43~100%)
Wu et al. [25], 2021, J Surg Oncol	Shanghai Ninth People’s Hospital, Shanghai Jiao Tong University School of Medicine	2014~2019	28 ^#^	48.1 ± 11.6	15/13	I + II (*n* = 10) I + II + III (*n* = 6) II + III (*n* = 4) II (*n* = 4) I (*n* = 4)	393 (220~600)	4404 (600~11,000)	Wide for 26; Marginal for 2	Superficial infection (*n* = 6) Hip dislocation (*n* = 3)	32.3 (3~75)	23.2 (17~29)
Wang et al. [26], 2020, Clin Orthop Relat Res	West China Hospital, Sichuan University	2016~2017	13	46 (31~66)	6/7	I + II (*n* = 3) I + II + III (*n* = 10)	260 (170~540)	2600 (900~8200)	Wide for all	Delayed wound healing (*n* = 2)	27 (24~31)	23 (15~27)
Wang et al. [27], 2018, Int Orthop	Union Hospital, Tongji Medical College, Huazhong University of Science and Technology	2015~2016	11	47 (21~63)	5/6	Not specified	271 ± 45.5	3236 ±1665	Wide for 9; Marginal for 2	Delayed wound healing (*n* = 1) Hip dislocation (*n* = 2)	15.5 (6~24)	19.2 (13~25)
Current study	Fudan University Shanghai Cancer Center	2018~2021	16	42.8 (19~67)	8/8	I + II (*n* = 5) II + III (*n* = 7) II (*n* = 3) I + II + IV (*n* = 1)	289.7 (200~390)	1563 (400 to 3600)	Wide for all	Deep venous thrombosis (*n* = 2) Pneumonia (*n* =2) Would infection (*n* =2) Hip dislocation (*n* = 2)	17.75 (6~46)	85.8% (26.7~100%) or 25.8 (8~30)

^#^ Only 24 patients underwent periacetabular tumor resection and reconstruction. Data are presented with mean or median with range or SD (standard deviation).

## Data Availability

The original data were available from the corresponding author upon an appropriate request.

## Supplementary Materials

The following supporting information can be downloaded at: https://www.mdpi.com/article/10.3390/bioengineering9080400/s1, Figure S1: Guiding plate for osteotomy.
