# Supplementary figures and images for: Computer-Aided Design and 3D Printing of Hemipelvic Endoprosthesis for Personalized Limb-Salvage Reconstruction after Periacetabular Tumor Resection
Source: Bioengineering (Basel). 2022 Aug 18;9(8):400. doi: 10.3390/bioengineering9080400 (PMC9405276; doi:10.3390/bioengineering9080400)

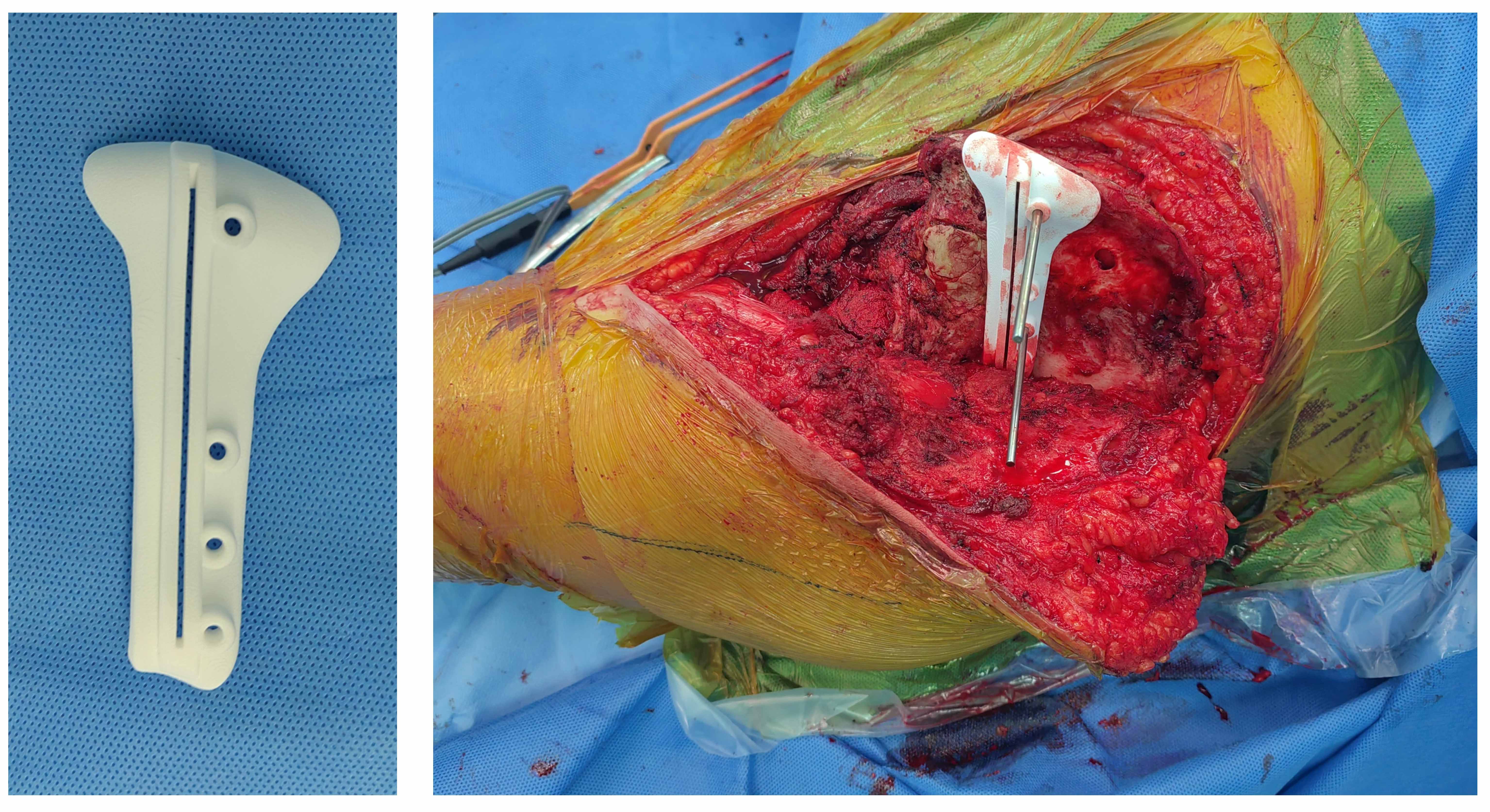

Supplement: Supplementary file 1 [file bioengineering-09-00400-s001.zip › Figure S1.jpg]
